# Winter Walk

**DOI:** 10.5811/westjem.58365

**Published:** 2023-03-22

**Authors:** Corey S. Hazekamp, Ryan N. Barnicle

**Affiliations:** * NYC H+H/Lincoln, Department of Emergency Medicine, Bronx, New York; † The Warren Alpert Medical School of Brown University, Department of Emergency Critical Care, Providence, Rhode Island

## Abstract

Winter Walk is a photo essay meant to be an inspirational commentary on emergency medicine’s role in meeting the needs of our most vulnerable patients. Oftentimes, the social determinants of health, now well reviewed in the modern medical school curriculum, become intangible concepts that get lost amongst the busy environment of the emergency department. The photos within this commentary are striking and will move readers in various ways. The authors hope that these powerful images generate a mix of emotion that ultimately motivates emergency physicians to embrace the emerging role in addressing the social needs of our patients both inside and outside the emergency department.

We are emergency physicians. We resuscitate the dead and dying, and then we keep them alive. We work up, clear, and discharge patients. We tidy up the board, sign out, and then go home. Right?

Historically, our scope of practice has been confined to the hustle and bustle of the emergency department (ED), and the appeal for many is the ability to walk away at the end of the shift. Things have changed though, and our specialty is evolving. The pandemic has codified our place on the frontline of population health. For many individual patients, as well as the greater public good, the ED is all that stands against the growing number of societal harms. This obligation does not end with infectious diseases but encompasses all the worsening public health crises: firearm deaths; homelessness; unmanaged mental health; and more. Embracing our role in addressing the needs of our local communities, such as the worsening opioid use disorder crisis, is now an essential part of our job. This truth may be no more evident than during a walk right outside the ED’s front door. As the saying goes, a picture speaks a thousand words…

On a frigid winter day with just a few minutes to spare, the opportunity to step outside during a busy shift presented itself. After turning the corner onto a familiar street, the sidewalk was found to be obstructed by piles of used syringes. There were unfolded cardboard boxes, empty food containers, and dirty clothes spilled across the sidewalk among the syringes. Community members, excluded from the photo, were actively using intravenous (IV) drugs as they huddled in a doorway within the brick wall of the hospital—as visible to the public as the sun’s light.[Fig f1-wjem-24-461]

These photos were taken in a neighborhood within the poorest congressional district of the United States. On one side of the street was a school, and on the other, the hospital. Here was a nearly impassable mess of used syringes and the remnants of a frozen winter campsite, juxtaposed between the two pillars of society meant to empower individuals to lead fuller lives. The painful irony of these images is demoralizing. There were emergency physicians working tirelessly to save the lives of patients inside, while just on the other side of the wall other patients were struggling to get by.[Fig f2-wjem-24-461]

Truthfully, most of us have probably never used IV drugs or even seen someone actively using them. We will probably never truly understand what life is like for our patients between injections. But we have all written “IVDU” (intravenous drug use) in a chart or formed a differential diagnosis after considering a patient’s drug use history. Then, we drain the soft tissue abscesses and schedule wound checks. We treat endocarditis with broad-spectrum antibiotics and admit for sepsis. We revive victims of accidental overdoses from a drug supply tainted with fentanyl, and we monitor for clinical sobriety. We know these complications well, but it is hard to gain true insight into the day-to-day lives of some of our most vulnerable patients. Perhaps these photos may represent a glimpse into these hardships.

After it is safe for a patient to leave the hospital, what are we doing next for them? What is the environment we are sending them to live in? Here, while visualizing what life looks like for some of our most vulnerable patients, ask yourself how these photos make you feel as a physician. Understandably, some will feel the despair of defeat as if the care we provide matters little if this is the inevitable result just outside our doors. Others will feel invigorated to redouble their efforts to ensure adequate follow-up for the patients they induce on buprenorphine during an upcoming shift.[Fig f3-wjem-24-461]

Pause. Take a moment and look closely. The details matter.

These needles are discarded after a clean single use, the empty boxes left behind as proof. There are filters and sterile saline. They have come from a local harm reduction program meant to mitigate the infectious mortality and morbidity associated with opioid use disorder. There are discarded fentanyl test strips and empty naloxone atomizers among the debris, too, likely given just on the other side of those brick walls meant to prevent the next lethal overdose. These are hopeful details, partially hidden within the instilling dread of this social blight but keenly evident to an observant eye.

This is not a battle lost, but a fight that has just begun. Physicians in all specialties are feeling the burden of new social imperatives, and the field of medicine as a whole is on its own so-called winter walk right now. Despite the current biting cold of a dysfunctional healthcare system, the embers of hope must be kindled. As emergency medicine evolves, it must continue to embrace the imperative needs of the most vulnerable. Our patients need us inside and outside the ED, and our specialty continues to rise to the occasion.

## Figures and Tables

**Figure 1 f1-wjem-24-461:**
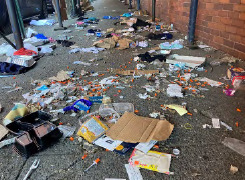
A photograph of a sidewalk just around the corner from an emergency department, strewn with empty boxes and used syringes.

**Figure 2 f2-wjem-24-461:**
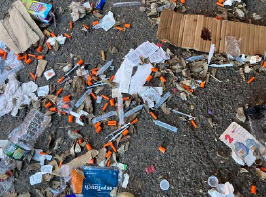
This seeming image of despair in fact reveals a roadmap toward hope, evidenced in needles discarded after a clean single use, used fentanyl test strips, and empty naloxone atomizers meant to prevent the next lethal overdose.

**Figure 3 f3-wjem-24-461:**
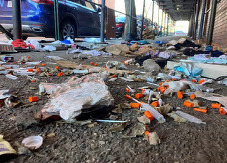
A snapshot of the hardships that some our most vulnerable patients face after discharge from the emergency department.

